# Interplay between monocyte subsets and leukemic blasts shapes the immune microenvironment and sustains minimal residual disease in pediatric B-cell acute lymphoblastic leukemia

**DOI:** 10.3389/fimmu.2026.1776564

**Published:** 2026-08-18

**Authors:** Linlin Zhang, Xiaoyang Liu, Fang Chen, Gang Xu, Yang Fan

**Affiliations:** 1 Department of Pediatrics, Shengjing Hospital of China Medical University, Shenyang, China; 2 Hematology Laboratory, Shengjing Hospital of China Medical University, Shenyang, China; 3 Medical Research Center, Shengjing Hospital of China Medical University, Shenyang, China

**Keywords:** cellular heterogeneity, immune microenvironment, minimal residual disease, monocytes, pediatric B-cell acute lymphoblastic leukemia, single-cell transcriptome sequencing

## Abstract

**Objective:**

Persistent minimal residual disease (MRD) significantly contributes to chemotherapy resistance and relapse in pediatric B-cell acute lymphoblastic leukemia (B-ALL), with conventional clinical risk factors insufficiently explaining the variability in patient prognoses. This study sought to explore potential mechanisms linked to sustained MRD by integrating analyses of clinical cohorts with single-cell transcriptomics, aiming to identify reliable biomarkers for risk stratification and targeted therapy.

**Methods:**

Ninety-seven children with newly diagnosed B-ALL were enrolled and stratified according to their end-of-induction (EOI)-MRD status. Survival analysis and regression models were employed to identify independent risk factors influencing clinical outcomes and MRD development. Single-cell sequencing and CellChat analysis were conducted on paired diagnostic and EOI bone marrow samples to characterize leukemic heterogeneity and the interactions between monocyte subsets and leukemic blasts.

**Results:**

Clinical survival analysis showed that pediatric B-ALL patients with positive EOI-MRD had significantly shorter event-free survival. Multivariate regression analysis identified patient age and conventional clinical risk stratification as independent prognostic factors for long-term clinical outcomes. Moreover, age, baseline risk grouping, and the elevated proportion of non-classical monocytes served as independent predictors of positive EOI-MRD status. Single-cell transcriptomic profiling revealed remarkable intratumoral heterogeneity in newly diagnosed pediatric B-ALL cases. Interferon (IFN)-responsive leukemic blasts constituted candidate drug-resistant clones associated with chemotherapy resistance and persistent MRD. This blast subset exhibited inherent malignant characteristics, including blocked B-cell differentiation, cell cycle dormancy, and adaptive IFN tolerance. These malignant clones displayed transcriptional signatures consistent with evasion of IFN-dependent anti-leukemic immune clearance and IFN signaling reprogramming under chemotherapy stress to support cell survival, which may correlate with the formation of residual leukemic lesions. Immune microenvironment analysis confirmed disrupted bone marrow immune homeostasis in MRD-positive patients, which was characterized by impaired T-cell immune surveillance and aberrant accumulation of non-classical monocytes. Global cell-cell communication analysis uncovered bidirectional crosstalk between leukemic blasts and monocytes in the B-ALL immune microenvironment. Leukemic blasts exerted predominant unidirectional regulation on monocytes, while monocytes reciprocally remodeled the malignant phenotypes of leukemic cells through multiple ligand-receptor interactions. Functional heterogeneity existed across different monocyte subsets in the leukemic niche. Classical and intermediate monocytes were the primary sources of CCL signaling, mediating chemotactic signals to leukemic blasts via the CCL axis. Galectin signaling was universally activated to support basal cell adhesion and niche crosstalk in the bone marrow microenvironment. In comparison, non-classical monocytes exhibited transcriptional signatures suggestive of pro-tumor activities via activation of TNF/TNFSF13B-dependent pro-inflammatory signaling and downstream VEGF-related vascular remodeling pathways, which may correlate with the persistence and progression of residual leukemia. Notably, despite intensive inflammatory crosstalk between non-classical monocytes and IFN-responsive residual leukemic blasts, non-classical monocytes only displayed compensatory upregulation of HLA-DRB5 without full transcriptional activation of inflammatory programs. This incomplete immune response may be unable to eradicate residual leukemic cells and favor the formation of a tumor-permissive bone marrow niche, potentially correlating with persistent MRD in pediatric B-ALL. In conclusion, clinical high-risk factors, intrinsic leukemic heterogeneity, and monocyte-associated immune disturbances are jointly correlated with MRD progression. Aberrant crosstalk between non-classical monocytes and leukemic cells is linked to chemotherapy resistance, providing candidate immune biomarkers for prognostic evaluation and personalized targeted therapy in pediatric B-ALL.

## 1 Introduction

Acute B-cell lymphoblastic leukemia (B-ALL) is the most prevalent hematological malignancy in children. Recent advances in standard chemotherapy protocols and the widespread adoption of personalized treatment strategies have significantly improved the long-term survival rates of pediatric patients with B-ALL. Nevertheless, despite the generally favorable prognosis, minimal residual disease (MRD) at the end of induction (EOI) remains the most significant independent risk factor for disease relapse and adverse clinical outcomes, posing a major barrier to improving the cure rates in high-risk pediatric patients (1, 2). A subset of children continue to harbor persistent MRD after conventional chemotherapy, indicating that traditional chemotherapy is effective only against actively proliferating leukemic cells. It fails to correct pathological abnormalities in the bone marrow microenvironment or eradicate the core mechanisms driving leukemic immune evasion and lesion persistence, ultimately leading to sustained MRD and disease recurrence.

Previous investigations into chemoresistance and MRD persistence in B-ALL have primarily focused on cell-intrinsic abnormalities of leukemic cells, including somatic gene mutations and the activation of drug efflux pumps (3, 4). However, with advances in tumor immune microenvironment research, accumulating evidence indicates that disruption of bone marrow immune homeostasis plays a critical role in leukemia progression, therapeutic resistance, and immune evasion (5). As integral components of this microenvironment, bone marrow immune cells undergo subset dysregulation and functional reprogramming, contributing to the establishment of an immunosuppressive niche that shelters leukemic cells and sustains MRD. While current research has predominantly focused on the adverse effects of T cell exhaustion and aberrant T cell subset distribution on B-ALL prognosis (6–9), the specific roles of monocyte subsets remain underexplored. Notably, the relationship between monocyte subpopulations and EOI-MRD positivity, along with their regulatory mechanisms, has yet to be systematically elucidated.

Monocytes constitute a highly heterogeneous population of cells, which are categorized into three primary subsets: classical monocytes (CD14^+^CD16^−^), intermediate monocytes (CD14^+^CD16^+^), and non-classical monocytes (CD14^low^CD16^+^). Non-classical monocytes are well-recognized for their pivotal functions in microenvironment reshaping and immunosuppressive modulation across multiple malignancies (7, 10, 11). Nevertheless, their prognostic significance and the molecular mechanisms underlying MRD persistence in pediatric B-ALL remain inadequately understood. In this study, we enrolled pediatric patients with newly diagnosed B-ALL and stratified them into MRD-positive and MRD-negative groups based on EOI-MRD status. We conducted a systematic comparison of the bone marrow T cell and monocyte subset profiles at diagnosis, with the objective of identifying key immune cell subsets associated with MRD outcomes. Clinical analyses indicated that EOI-MRD positivity correlated with altered T-cell subset repertoire and prominent expansion of CD14^low^CD16^+^ non-classical monocytes. The aberrant accumulation of CD14^low^CD16^+^ non-classical monocytes within the bone marrow has been identified as an independent adverse prognostic factor for EOI-MRD positivity, implying that this monocyte subset facilitates MRD formation via constructing an immunosuppressive bone marrow microenvironment to support leukemic cell survival.

To further investigate the molecular mechanisms through which CD14^low^CD16^+^non-classical monocytes sustain persistent MRD, we conducted single-cell RNA sequencing to characterize the transcriptomic landscapes of bone marrow cells from both MRD-positive and MRD-negative patients at diagnosis and EOI. In conjunction with an analysis of leukemic blast heterogeneity, our findings revealed that distinct leukemic subset distribution patterns and the retention of chemotherapy-resistant leukemic clones are strongly correlated with MRD persistence. Furthermore, extensive crosstalk was observed between leukemic cells and monocyte subsets within the bone marrow microenvironment. Non-classical monocytes exhibited signatures associated with activated downstream pro-survival signaling, which may favor leukemic immune evasion and persistent lesion retention in pediatric B-ALL.

In conclusion, this study comprehensively illustrates the potential role of non-classical monocytes in sustaining leukemic persistence in pediatric B-ALL, through clinical correlation analysis, cellular phenotyping and single-cell molecular profiling. Our findings provide potential biomarkers for MRD-related risk stratification and novel theoretical evidence for targeted therapeutic intervention in pediatric B-ALL.

## 2 Materials and methods

### 2.1 Study subjects and screening criteria

This retrospective clinical study consecutively enrolled 98 pediatric patients with newly diagnosed B-ALL who received standardized treatment at the Department of Pediatric Hematology, Shengjing Hospital of China Medical University, from January 2022 to June 2024. The median age of the enrolled children was 4 years (range, 1.25–13 years). According to bone marrow immunophenotyping results, the cohort consisted of 9 cases of pro-B ALL, 26 cases of pre-B ALL, and 62 cases of common-B ALL. Among them, one patient with common-B ALL presented an increased proportion of leukemic blasts with monocyte-like morphology, and one patient showed aberrant myeloid antigen expression on leukemic cells. The inclusion criteria were as follows: patients with newly diagnosed primary B-ALL, complete clinical and follow-up data, and eligibility for standardized treatment with the CCLG-2008 protocol. The exclusion criteria included patients with other concomitant hematological malignancies, recurrent B-ALL, or prior antitumor treatment before admission; patients with severe congenital malformations, vital organ dysfunction, or other serious underlying diseases that precluded completion of standardized chemotherapy; cases with missing clinical, laboratory, or follow-up information; and patients who voluntarily discontinued treatment or were lost to follow-up. All initially enrolled patients were scheduled to receive standardized chemotherapy based on the CCLG-2008 protocol (12). Of the 98 initially enrolled patients, one child died of severe infection during induction therapy and was unable to complete subsequent treatment and follow-up, and was therefore excluded. Ultimately, 97 patients were included in the final analysis. Written informed consent was obtained from the guardians of all enrolled children. This study was approved by the Ethics Committee of Shengjing Hospital of China Medical University (Approval No. 2025PS1472K) and conducted in strict accordance with the principles of the Declaration of Helsinki.

Based on the same study period and unified screening criteria, six pediatric patients with newly diagnosed B-ALL were further enrolled to form a subcohort for single-cell RNA sequencing (scRNA-seq), aiming to deeply analyze the bone marrow immune microenvironment. Bone marrow samples were collected at two key time points: diagnosis (Dx) and EOI. According to the EOI-MRD status, the subcohort was divided into the MRD-positive group (MRD+, n=3) and the MRD-negative group (MRD-, n=3). All patients in the subcohort met the established inclusion and exclusion criteria. The scRNA-seq part of this study was approved by the Ethics Committee of Shengjing Hospital of China Medical University (Approval No. 2022PS468K).

### 2.2 Sample collection and preprocessing

EDTA-anticoagulated bone marrow samples were collected from all enrolled pediatric patients for subsequent targeted gene sequencing, flow cytometric immunophenotyping, and MRD quantification. For patients in the scRNA-seq subcohort, bone marrow samples were harvested at diagnosis and EOI. Bone marrow mononuclear cells were isolated via density gradient centrifugation, washed with Dulbecco’s Phosphate Buffered Saline (DPBS) to remove cell debris, and stored long-term in liquid nitrogen until use. After thawing, the cells were purified through centrifugation and filtration to ensure cell viability met the standards for single-cell library construction.

### 2.3 Targeted gene sequencing and RNA sequencing

Genomic DNA (gDNA) was isolated from bone marrow or peripheral blood specimens using the MagCore Genomic DNA Whole Blood Kit (Concert, China), following the manufacturer’s protocols. The concentration and quantity of extracted gDNA were determined with a Qubit 4.0 Fluorometer and quantitative real-time PCR (both Thermo Fisher Scientific, USA), respectively. Subsequently, 300 ng of fragmented gDNA was used to construct sequencing libraries with the KAPA DNA Hyper Prep Kit (Roche, Switzerland). A custom 245-gene targeted sequencing panel was provided by Acornmed Biotechnology Co., Ltd. All DNA libraries were sequenced on the DNBSEQ-T7 platform (BGI Genomics, China). Raw variant data were filtered according to the following criteria: mean on-target effective sequencing depth no less than 1000×, mapping quality (MQ) no less than 30, base quality (BQ) no less than 30; the variant allele frequency (VAF) was set to no less than 0.8% for single nucleotide variations (SNVs) and small insertions and deletions (InDels). Trimmed sequencing reads were aligned to the reference genome using Burrows-Wheeler Aligner (BWA, v0.7.12). PCR duplicates were marked via the MarkDuplicates tool in Picard. The IndelRealigner and BaseRecalibrator modules of the Genome Analysis Toolkit (GATK, v3.8) were applied for read realignment and base quality recalibration, respectively. SNVs and small InDels were called using Mutect2. All genetic variants were functionally annotated with ANNOVAR, incorporating data from the 1000 Genomes Project, COSMIC, SIFT and PolyPhen databases.

Total RNA was extracted from bone marrow or peripheral blood cells using the PAXgene^®^ Blood RNA Kit (Qiagen, Germany). RNA integrity and concentration were assessed using an Agilent 2100 Bioanalyzer (Agilent Technologies, USA) and Qubit (Life Technologies, USA). RNA samples meeting the following criteria were included: RNA integrity number (RIN) no less than 6.0, no obvious degradation, and total RNA yield no less than 500 ng. RNA-seq libraries were generated with the Watchmaker RNA Library Preparation Kit (WatchMaker, USA) and sequenced on the MGISEQ-T7 platform (MGI, China). Reads with a Q30 ratio no less than 85% were retained for subsequent analysis. Adapter sequences and low-quality reads were removed using fastp (v0.19.3). The resulting clean reads were mapped to the human reference genome GRCh37 via STAR (v2.7.10a). Gene fusion events were detected using Arriba (v2.3.0).

### 2.4 Flow cytometric immunophenotyping and minimal residual disease detection

Bone marrow samples (2 mL) were collected from newly diagnosed B-ALL patients and anticoagulated with heparin. After enumeration of bone marrow nucleated cells, the cell concentration was adjusted to (1×10^6^) cells/mL to prepare cell suspensions for flow cytometric analysis. All fluorescently conjugated monoclonal antibodies were obtained from BD Biosciences and Beckman Coulter (USA) for immunophenotyping of T cell and monocyte subsets. The T-cell antibody panel consisted of CD3-PC7, CD4-ECD, CD8-PC5.5, CD28-FITC, PD-1 (CD279)-APC, CTLA-4 (CD152)-PE and CD45-APC-A750. The monocyte subset panel included CD14-PE, CD16-FITC, CD45-PerCP and CD33-APC.

Two 8-color pre-formulated antibody panels were applied for flow cytometric MRD detection, with all antibodies sourced from Beckman Coulter and BD Biosciences (USA).

Panel 1: CD20-FITC/CD81-PE/CD10-ECD/CD13-PC5.5/CD19-PC7/CD34-APC/CD38-APC-A700/CD45-APC-A750;
Panel 2: CD58-FITC/CD22-PE/CD10-ECD/CD33-PC5.5/CD19-PC7/CD34-APC/CD38-APC-A700/CD45-APC-A750.

In brief, approximately 1×10^6^ bone marrow nucleated cells per tube were incubated with 70 μL pre-mixed antibodies. After vortexing, samples were kept in the dark at room temperature for 15 minutes. Subsequently, 500 μL red blood cell lysis buffer was added, and samples were incubated for another 10 minutes in the dark to lyse erythrocytes. Following a single wash with normal saline, all samples were acquired on a flow cytometer within 1 hour of sample preparation.

### 2.5 Flow cytometer acquisition and data analysis

All samples were acquired using a Beckman Coulter DxFlex flow cytometer, and data analysis was performed with Kaluza Analysis software. A strict sequential gating strategy was applied for all flow cytometric analyses. Briefly, events were first gated on forward scatter-area (FSC-A) versus side scatter-area (SSC-A) to identify intact nucleated cells and exclude cellular debris, and single cells were further discriminated using FSC-H versus FSC-A to remove cell aggregates. CD45 expression combined with SSC parameters was then used to separate lymphocytes, monocytes, and granulocyte populations. For T-cell phenotyping, lymphocytes were gated within the CD45-positive lymphocyte cluster, and CD3^+^ T cells were further subdivided into CD4^+^ and CD8^+^ subpopulations. Fluorescence minus one (FMO) controls were specifically applied to define positive expression thresholds for CD28, CD152 (CTLA-4), and CD279 (PD-1) in T-cell subsets to eliminate non-specific background fluorescence interference. For monocyte subset analysis, monocytes were identified as CD45^+^CD33^+^ populations and further categorized into three subsets according to CD14 and CD16 expression levels: classical monocytes (CD14^+^CD16^−^), intermediate monocytes (CD14^+^CD16^+^), and non-classical monocytes (CD14^low^CD16^+^). For MRD measurement, at least 500,000 events were collected for each sample to ensure sufficient analytical sensitivity and reliable detection results.

### 2.6 Single-cell library construction and high-throughput sequencing

Single-cell capture, reverse transcription, cDNA amplification, and sequencing library construction of bone marrow mononuclear cells were performed using the BD Rhapsody high-throughput single-cell mRNA sequencing platform. Single cells were randomly loaded into more than 200,000 independent microwells via limiting dilution, followed by adequate bead loading to achieve one-to-one pairing between individual cells and capture beads. After in-well cell lysis, polyA-tailed mRNA transcripts were hybridized to polyT sequences on the capture beads. All beads were harvested and pooled for subsequent reverse transcription and Exonuclease I (ExoI) digestion. Each captured cDNA molecule was tagged with a unique molecular identifier (UMI) and a cell barcode at the 3′ end. Full-length transcriptome libraries were constructed according to the standard BD Rhapsody whole-transcriptome amplification (WTA) workflow, including random priming and extension (RPE), RPE amplification PCR, and WTA indexing PCR. Library quantification and quality assessment were performed using the Agilent 4150 TapeStation System with High-Sensitivity D1000 ScreenTape, as well as the Qubit High-Sensitivity DNA Assay Kit (Thermo Fisher Scientific). Qualified libraries were sequenced on the Illumina NovaSeq platform with 150 bp paired-end reads. Raw sequencing data were processed using the BD Rhapsody WTA Analysis Pipeline (v1.8) based on the human reference genome GRCh38 and GENCODE v32 annotations. The official pipeline initially converted raw FASTQ files into UMI count matrices. Raw reads were subjected to quality control with FastQC, followed by barcode demultiplexing and UMI deduplication prior to the generation of final expression matrices. All sequencing procedures and preliminary bioinformatic preprocessing were performed by Genechem Co., Ltd. (Shanghai, China).

### 2.7 Single-cell data preprocessing and batch correction

Raw single-cell transcriptomic data were processed with the Seurat v4 package in R. The raw UMI count matrix generated by the official BD pipeline was initially pre-filtered and normalized in Python using Scanpy (v1.8). For the first round of quality control (QC), low-quality cells and potential multiplets were preliminarily excluded according to predefined thresholds: cells with total UMI counts greater than 100,000, detected gene numbers greater than 8,000, or a mitochondrial gene expression ratio exceeding 25% were excluded. The dataset was normalized globally with a scaling factor of 10,000 and log-transformed subsequently. Highly variable genes were detected following standard protocols. We performed principal component analysis (PCA) and constructed cell neighbor graphs for clustering. Uniform Manifold Approximation and Projection (UMAP) was adopted to visualize cells in two and three dimensions. Datasets passing preliminary QC in Scanpy were imported into Seurat for downstream analysis. DoubletFinder (v2.0.3) was used to eliminate potential doublets under default settings. A second round of stringent QC was conducted: only cells harboring 200–6,000 detected genes, mitochondrial gene ratio below 20% and reasonable UMI counts were retained to remove cell debris and residual doublets. The Harmony algorithm was applied to remove inter-sample batch effects and integrate all datasets. The integrated data were normalized and log-transformed using NormalizeData. The top 2000 highly variable genes were identified via FindVariableFeatures, and ScaleData was used for standardization. The top 30 principal components were selected for dimensionality reduction, cell clustering and monocyte subpopulation analysis.

### 2.8 Fine annotation of monocyte subsets and copy number variation analysis with subtype identification of B-lineage leukemic cells

Regularized principal component analysis (RPCA) was performed for dimensionality reduction of the integrated dataset, and unsupervised clustering was used for preliminary cell classification. Initial cell annotation was completed based on differentially expressed genes and canonical lineage markers. To distinguish malignant leukemic cells from residual normal hematopoietic cells, copy number variation (CNV) analysis was conducted using the InferCNV v3 algorithm, with endogenous normal T cells serving as internal references to identify malignant genomic characteristics of leukemic cells. Based on the CNV profiles, B-lineage cells were re-clustered and finely annotated to screen functionally distinct leukemic subtypes, and the molecular characteristics of each subtype were validated according to the expression patterns of signature genes.

For precise immune cell annotation, single-cell transcriptomic data were used for preliminary lineage identification of bone marrow cells. Myeloid cells were further extracted for secondary fine clustering, with dendritic cells, macrophages and other contaminating cells excluded. Combined with canonical marker genes including *CD14, FCGR3A, S100A8, LYZ*, and *CX3CR1*, three monocyte subsets were accurately annotated, namely classical monocytes, intermediate monocytes, and non-classical monocytes.

Moreover, healthy bone marrow single-cell datasets from two healthy donors retrieved from published literature were integrated to construct a reference atlas covering full hematopoietic lineages. Patient single-cell datasets were projected onto the UMAP space of the established reference atlas, and MapQuery-based label transfer was performed to further confirm the cell identity of normal hematopoietic cells and malignant B-lineage blasts.

### 2.9 Cell communication analysis between monocyte subsets and leukemic cells

CellChat was used to explore the interaction and regulatory mechanisms between the three monocyte subsets and leukemic blasts within the bone marrow immune microenvironment. Based on well-annotated ligand-receptor databases, we quantitatively analyzed the strength and direction of intercellular signaling crosstalk between each monocyte subset and leukemic cells, with a focus on ligand-receptor expression patterns specific to non-classical monocytes. Pathway enrichment analysis was conducted to identify core signaling pathways, including GALECTIN, TNF, CCL and VEGF pathways. This analysis aimed to clarify how non-classical monocytes reshape the bone marrow immune microenvironment via cell-cell crosstalk and thereby facilitate the persistence of minimal residual disease in B-ALL.

### 2.10 Statistical analysis

Clinical data were analyzed using SPSS 27.0.1. For quantitative variables, normally distributed data were expressed as mean ± standard deviation and compared using independent sample t-tests, while non-normally distributed data were presented as median (interquartile range) and compared via the Mann-Whitney U test. Categorical variables were described as cases (percentages), and intergroup differences were evaluated using the chi-square test. For prognostic assessment, event-free survival (EFS) was defined as the time from initial diagnosis to induction failure, disease recurrence, all-cause death, or secondary malignancy. Kaplan-Meier survival curves were plotted, and the log-rank test was used to compare survival differences between groups. Multivariate Cox regression analysis was performed to identify independent prognostic factors for EFS. Univariate and multivariate binary logistic regression models were further used to screen independent risk factors for positive EOI-MRD. For single-cell transcriptomic analyses, the Benjamini–Hochberg false discovery rate (FDR) correction was applied to all multiple comparative tests, including differentially expressed gene (DEG) screening, gene ontology (GO) pathway enrichment, and quantitative comparisons of immune and leukemic blast subsets. Genes with adjusted P (p.adjust) < 0.05 and |log2FC| > 0.5 were identified as significantly differentially expressed. Simple exploratory subgroup stratification analyses of rare immune cell populations were not adjusted for multiple testing, and related preliminary observations require further verification in larger independent patient cohorts. Statistical analysis and visualization of single-cell transcriptomic data were conducted using R software. Quantitative single-cell data were presented as mean ± standard error of the mean (SEM). Two-group comparisons were performed using independent sample t-tests, and multiple-group comparisons were analyzed by one-way ANOVA. All statistical tests were two-tailed, and an adjusted P value (FDR) less than 0.05 was considered statistically significant.

## 3 Results

### 3.1 Clinical and molecular characteristics, prognosis, and immune factors associated with MRD positivity in pediatric patients

**Figure 1:**
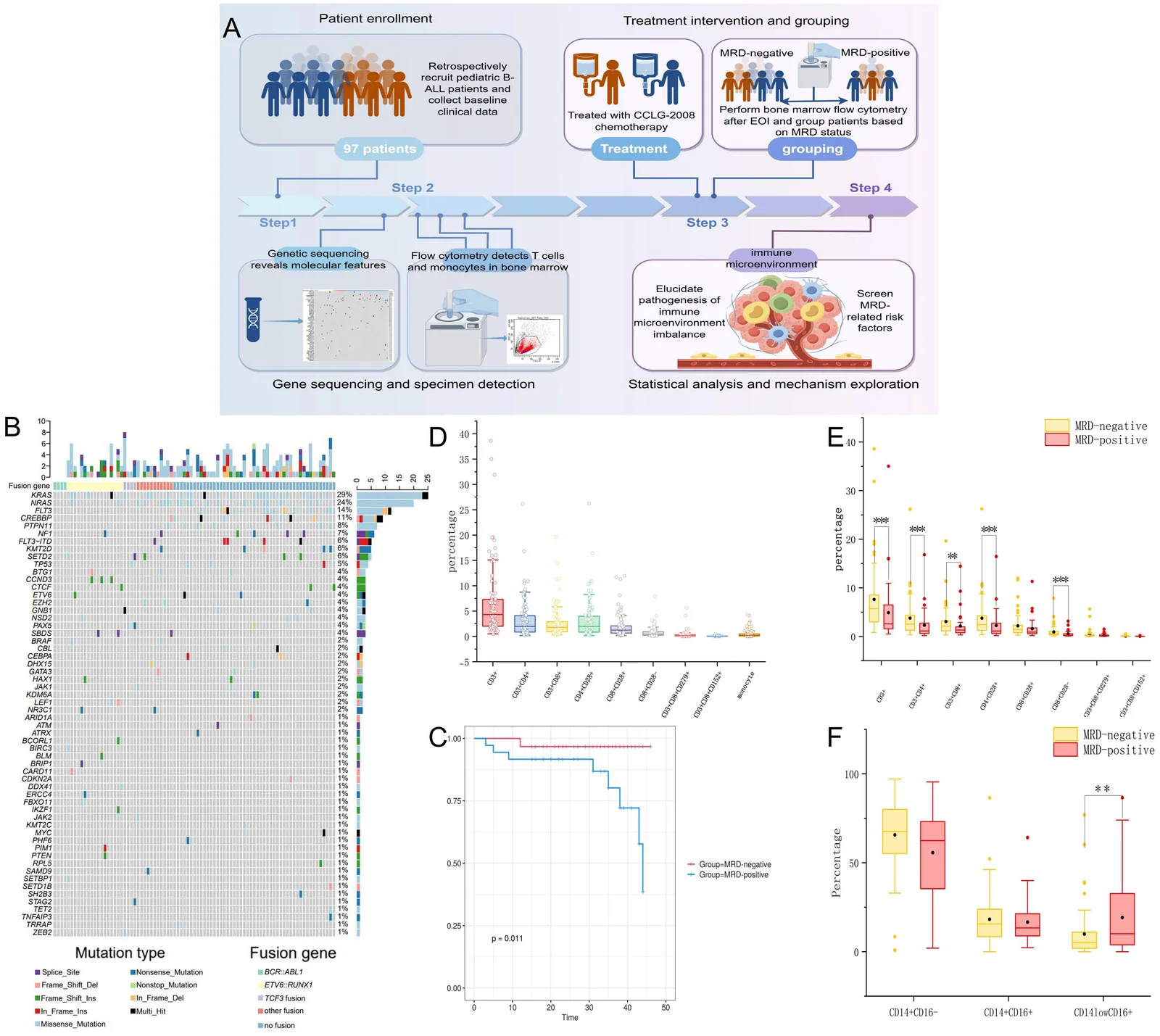
Study overview and multi-dimensional analysis of pediatric B-cell acute lymphoblastic leukemia. **(A)** Schematic workflow of the present study, including patient screening, clinical treatment, stratification based on end-of-induction minimal residual disease (EOI-MRD) status, and multi-omics analyses including genetic sequencing and immune phenotyping. **(B)** Genetic mutation landscape and fusion gene classification of the 97 enrolled patients. **(C)** Kaplan-Meier curves of event-free survival (EFS) stratified by EOI-MRD status (log-rank P = 0.011). **(D)** Distribution of immune cell subsets at initial diagnosis. **(E)** Comparison of T cell subsets between MRD-negative and MRD-positive groups. **(F)** Comparison of monocyte subsets between the two groups. All quantitative data are presented as box-and-whisker plots. *P < 0.05, **P < 0.01, ***P < 0.001.

The overall study workflow is depicted in **Figure 1A**. Initially, 98 children with newly diagnosed B-ALL were enrolled. One patient died of severe infection during induction therapy and could not complete subsequent treatment and follow-up, leaving 97 patients for the final analysis. All participants underwent comprehensive baseline assessments at diagnosis, including demographic data, bone marrow blast counts, genetic profiling and immunophenotyping. All patients achieved morphological complete remission following induction therapy administered per the CCLG-2008 protocol (12). EOI-MRD was detected by flow cytometry at the end of the 4-week induction chemotherapy cycle (the predefined time point for MRD evaluation in the CCLG-2008 protocol), with a minimum detectable sensitivity of 0.01% leukemic blasts for this assay. Patients were grouped based on the uniform cutoff value of 0.01% EOI-MRD: those with detectable residual blasts < 0.01% were defined as the MRD-negative group, while patients with EOI-MRD ≥ 0.01% were categorized into the MRD-positive group. In total, 61 patients were MRD-negative and 36 were MRD-positive according to the above grouping criteria. Baseline comparisons (**Table 1**) revealed that patients in the MRD-positive group were significantly older and had higher absolute neutrophil counts relative to MRD-negative counterparts (all P less than 0.05). No significant intergroup differences were observed for other baseline clinical parameters.

**Table 1:** Clinical characteristics of 97 B-ALL patients stratified by minimal residual disease (MRD) status at the end of induction (EOI).

Parameters	Total	EOI		P-value
		MRD-negative	MRD-positive	
Median age(year)	4(1.25-13)	3.0(1.25-12)	7(2.08-13)	0.000021
Male to female	56/41	33/28	23/13	0.346
Leukocyte(*10^9/L)	9.2(1.1-441.3)	8.0(1.1-441.3)	15.9(2.3-403.8)	0.119
Neutrophils(*10^9/L)	0.7(0-13.2)	0.6(0-10.4)	1.2(0.1-13.2)	0.044
Lymphoctyes(*10^9/L)	4.2(0.3-91.2)	3.9(0.3-57.5)	4.6(1.3-91.2)	0.319
Monocytes(*10^9/L)	0.8(0-25.6)	0.7(0-21.2)	1.0(0-25.6)	0.717
Eosinophils(*10^9/L)	0(0-2.3)	0(0-1.5)	0(0-2.3)	0.419
Basophils(*10^9/L)	0(0-4.5)	0(0-4.5)	0(0-0.5)	0.984
Hemoglobin(g/l)	78(21-127)	76(21-127)	81.5(41-124)	0.536
Platelet(*10^9/L)	47(5-612)	48(6-612)	41(5-511)	0.709
Absolute Blast Count(*10^9/L)	2.6(0-397.2)	2.5(0-397.2)	5.1(0-363.4)	0.351
Lansky score(≥70points)	79	49	30	0.713
Lansky score(<70points)	18	12	6	–
Bone Marrow Blast Percentage(%)	89.2(7.6-97.6)	89.6(7.6-96.4)	89.2(48-97.6)	0.426
Lactate Dehydrogenase(U/L)	454(18.7-4557)	429(173-4146)	501(18.7-4557)	0.235
Central Nervous System(CNS1,CNS2, CNS3)	86/8/3	54/6/0	32/2/3	0.062
NCCN Risk Stratification Based on Cytogenetics Favorable/Intermediate/poor	29/64/4	19/40/2	10/24/2	0.829
Comprehensive disease risk stratification Favorable/Intermediate/poor	31/40/26	28/24/9	3/16/17	0.000073

All data represent baseline clinical information of patients at initial diagnosis. Data are presented as median (range: minimum-maximum). A p-value of less than 0.05 was deemed statistically significant and is highlighted in bold.

To delineate molecular subtypes and mutational landscapes across the cohort, targeted leukemia sequencing and whole-transcriptome sequencing were performed on bone marrow samples from 85 patients. Based on fusion gene patterns, all cases were categorized into five molecular subgroups: *ETV6-RUNX1* (n = 17), *BCR-ABL1* (n = 4), *TCF3* rearrangement (n = 4), other rare fusions (n = 11), and fusion-negative cases (n = 49). The full mutational spectrum is presented in **Figure 1B**. Recurrent driver mutations identified in this cohort included *KRAS, NRAS, FLT3* and *CREBBP.* The remaining 12 patients received only routine fusion gene screening: *ETV6-RUNX1* was detected in two cases and *MLL-AF9* in one case, whereas no recurrent fusion genes were identified in the other nine individuals.

Long-term prognostic analyses were conducted using complete follow-up data from all 97 patients. The median follow-up duration was 42 months (range, 1–48 months). A total of 10 EFS events were documented, corresponding to an overall event rate of 10.3%. Kaplan-Meier survival analysis showed that patients with EOI-MRD positivity had significantly shorter EFS compared with MRD-negative patients (log-rank P = 0.011), indicating that EOI-MRD status is strongly linked to long-term prognosis in pediatric B-ALL. Given the limited number of EFS events (n=10) in this cohort, the multivariate Cox regression analysis was designated as exploratory, with results interpreted conservatively to mitigate potential overfitting and statistical bias. Prior to model construction, comprehensive diagnostic tests were performed to validate model fitness and robustness. The scaled Schoenfeld residual analysis was adopted to examine the proportional hazards assumption, and no remarkable time-dependent trends were detected for any covariates, verifying satisfactory compliance with the proportional hazards assumption. Additionally, variance inflation factor (VIF) analysis was utilized to assess multicollinearity across all covariates, with focused evaluation on the correlation between EOI-MRD status and disease risk stratification. All included covariates yielded VIF values ranging from 1.024 to 1.635, all below the threshold of 2.5, confirming the absence of significant multicollinearity in the regression model. A forced-entry multivariate Cox regression model was applied to simultaneously adjust for five predefined clinically critical confounding covariates. All categorical variables were analyzed with clear reference categories, and full model results including HR values, 95% CIs, and P values for all covariates were systematically presented in the revised multivariate survival analysis table (**Table 2**). The multivariate regression results revealed that EOI-MRD positivity did not serve as an independent prognostic factor for EFS (HR = 1.483, 95% CI: 0.271–8.111, P = 0.649). In contrast, comprehensive disease risk stratification (patients with intermediate and poor risk were combined into a single group for analysis, with favorable risk set as the reference) emerged as the sole independent predictor of adverse long-term EFS outcomes (HR = 7.571, 95% CI: 1.702–33.683, P = 0.008). The inconsistent prognostic performance of EOI-MRD between univariate and multivariate analyses is most likely attributable to the small number of EFS events, which resulted in limited statistical power and relatively unstable parameter estimation in the multivariate Cox model. Despite the loss of independent prognostic significance in the multivariate adjustment, the significant predictive effect of EOI-MRD in Kaplan–Meier analysis still supports its clinical value in evaluating early therapeutic response and preliminary risk stratification. Given the inherent statistical limitations of the low-event cohort in this exploratory analysis, further large-scale, prospective cohorts with sufficient EFS events and prolonged follow-up are urgently required to validate and consolidate the independent prognostic efficacy of EOI-MRD in pediatric B-ALL.

**Table 2:** Multivariate Cox proportional hazards regression model for event-free survival (EFS).

Variables	Coding & reference category	HR	95% CI	P value
Age at diagnosis (continuous)	Per 1-year increase	1.147	0.926–1.423	0.201
Initial white blood cell count (continuous)	Per 1×10^9^/L increase	0.966	0.872–1.071	0.529
Central nervous system involvement	Reference: CNS1; vs. CNS2/CNS3	0.610	0.158–2.353	0.472
Bone marrow blast percentage (continuous)	Per 1% increase	1.025	0.981–1.071	0.271
Comprehensive disease risk stratification	Reference: Favorable risk; vs. Intermediate/Poor risk	7.571	1.702–33.683	0.008
EOI-MRD status	Reference: MRD-negative; vs. MRD-positive	1.483	0.271–8.111	0.649

Study and Model Specifications: Total cohort = 97 pediatric B-ALL patients, total EFS events = 10; Model building strategy: forced simultaneous entry of all predefined clinical covariates; Proportional hazards (PH) assumption was validated via scaled Schoenfeld residual analysis with no time-dependent trends observed; No significant multicollinearity existed among all covariates (VIF range: 1.024–1.635, all VIF < 2.5).

EFS, event-free survival; HR, hazard ratio; CI, confidence interval; EOI-MRD, end-of-induction minimal residual disease; VIF, variance inflation factor.

Due to the limited number of EFS events (n=10) in this cohort, the multivariate Cox regression model was defined as exploratory to avoid overfitting and ensure robust statistical interpretation. All covariates were included in the model via forced-entry method without variable screening.

To explore the potential correlation between bone marrow immune microenvironment disturbance and MRD persistence, flow cytometry was performed to compare the distributions of T cell and monocyte subsets in bone marrow samples between the two groups (**Table 3**). Immune subset analysis showed that MRD-positive patients had significantly lower proportions of total CD3^+^ T cells, CD3^+^CD4^+^ helper T cells, CD3^+^CD8^+^ cytotoxic T cells, CD4^+^CD28^+^ activated T cells, and CD8^+^CD28^−^ inactive cytotoxic T cells (P less than 0.05). In contrast, the proportion of CD14^low^CD16^+^ non-classical monocytes was markedly elevated in the MRD-positive group (P = 0.018). This immune subset imbalance indicated altered T-cell compartment configuration and skewed monocyte subset profiling in MRD-positive patients. Such phenotypic shifts may remodel the leukemic immune microenvironment, facilitate leukemic immune escape, and ultimately contribute to sustained MRD. No significant differences were found in other immune cell subsets between groups. Of note, the immune analyses in this study were exploratory without multiple testing correction, and the conclusions need further verification in larger clinical cohorts.

**Table 3:** Statistical analysis of the proportions of various T-cell and monocyte immunophenotypes (Expressed as a percentage of nucleated cells) between MRD-positive and MRD-negative cohorts at the end of induction (EOI) in a sample of 97 B-ALL patients.

Immune cell subset	Total (n=97)	MRD-negative (n=61)	MRD-positive (n=36)	P-value	Effect size r
CD3^+^ T cells	4.32 (1.85, 7.62)	5.72 (2.86, 8.58)	2.46 (1.55, 6.51)	**0.003**	0.30
CD3^+^CD4^+^ T cells	1.97 (0.76, 3.73)	2.54 (1.24, 4.69)	1.13 (0.59, 2.77)	**0.003**	0.30
CD3^+^CD8^+^ T cells	1.80 (0.85, 2.96)	2.10 (1.15, 3.51)	1.24 (0.77, 1.99)	**0.011**	0.26
CD4^+^CD28^+^ T cells	1.97 (0.76, 3.69)	2.42 (1.23, 4.63)	1.11 (0.59, 2.76)	**0.003**	0.30
CD8^+^CD28^+^ T cells	1.25 (0.54, 2.08)	1.42 (0.76, 2.32)	0.84 (0.54, 1.80)	0.052	0.20
CD8^+^CD28^−^ T cells	0.44 (0.20, 0.82)	0.53 (0.31, 1.02)	0.23 (0.16, 0.55)	**0.001**	0.33
CD3^+^CD8^+^CD279^+^	0.18 (0.06, 0.31)	0.21 (0.08, 0.35)	0.10 (0.05, 0.23)	0.090	0.17
CD3^+^CD8^+^CD152^+^	0.03 (0.02, 0.08)	0.04 (0.02, 0.11)	0.03 (0.02, 0.05)	0.072	0.18
Bone marrow monocytes	0.21 (0.08, 0.52)	0.23 (0.09, 0.63)	0.17 (0.08, 0.36)	0.372	0.09
CD14^+^CD16^−^ monocytes	66.67 (52.63, 78.42)	67.63 (54.77, 80.10)	62.43 (34.37, 73.97)	0.061	0.19
CD14^+^CD16^+^ monocytes	14.80 (8.69, 22.46)	15.63 (8.38, 24.07)	13.39 (8.91, 21.51)	0.565	0.06
CD14^low^CD16^+^ monocytes	6.25 (2.12, 14.85)	5.00 (1.65, 11.02)	10.09 (3.69, 33.03)	**0.018**	0.24

All data were collected at the time of initial diagnosis. Data are presented as medians with interquartile ranges (IQR). Between-group comparisons were performed using the Mann-Whitney U test. Effect size r was calculated as |Z|/√N. This is an exploratory analysis and no correction for multiple comparisons was applied. Bold values indicate p‑values < 0.05, which were deemed statistically significant.

Given the significant differences in immune cell subsets between groups, regression analyses were applied to further verify the predictive value of immune indicators for EOI-MRD positivity. Univariate binary logistic regression analysis demonstrated that the proportion of CD14^low^CD16^+^ non-classical monocytes was positively correlated with EOI-MRD positivity (OR = 1.030, 95% CI: 1.004-1.056, P = 0.023), whereas no significant correlations were observed between T cell subsets and MRD status. To control for clinical confounding factors, non-classical monocyte proportion, age, disease risk stratification, and neutrophil count were incorporated into the multivariate logistic regression model. The results confirmed that increased CD14^low^CD16^+^ non-classical monocyte proportion (OR = 1.041, 95% CI: 1.008-1.075, P = 0.014), advanced age (OR = 1.354, 95% CI: 1.120-1.636, P = 0.002), and high-risk stratification (OR = 6.647, 95% CI: 2.623-16.849, P less than 0.001) were independent risk factors for EOI-MRD positivity, while neutrophil count had no independent predictive effect (P = 0.943).

In conclusion, EOI-MRD positivity in pediatric B-ALL is closely associated with bone marrow immune microenvironment disorder characterized by T cell immune suppression and aberrant enrichment of CD14^low^CD16^+^ non-classical monocytes. The proportion of CD14^low^CD16^+^ non-classical monocytes, age, and disease risk stratification are independent risk factors for post-induction MRD occurrence. Survival analyses indicated that MRD positivity serves as a valuable univariate indicator for adverse long-term prognosis, while disease risk stratification is the core independent factor affecting long-term survival. The present findings demonstrate that abnormal bone marrow immune microenvironment is a critical contributor to MRD persistence after induction therapy in pediatric B-ALL. These specific immune biomarkers provide reliable experimental evidence and clinical references for therapeutic response evaluation, recurrence risk prediction, and individualized treatment strategy formulation.

### 3.2 Single-cell sequencing reveals B-lineage cell heterogeneity and molecular mechanisms underlying MRD persistence in pediatric B-ALL

The single-cell sequencing subcohort consisted of 6 children with newly diagnosed B-ALL. Bone marrow samples were collected at diagnosis (Dx) and at the end of induction therapy. Participants were divided into the MRD-positive group (MRD+, n = 3) and MRD-negative group (MRD−, n = 3) based on EOI-MRD status. Detailed clinical information of these patients is summarized in **Supplementary Table 1**. To enable accurate cell annotation, we constructed a standardized reference atlas of normal bone marrow by integrating public single-cell datasets derived from 2 healthy donors (8). All single-cell transcriptomic data underwent rigorous quality control (QC), including filtering by detected gene numbers, unique molecular identifier (UMI) counts and mitochondrial gene expression ratios. Doublets and low-quality cells were removed accordingly. The QC outcomes were robust and reliable (**Supplementary Figure 2**). The Harmony algorithm was applied to correct batch effects, and a high-quality integrated dataset was finally generated.

**Figure 2:**
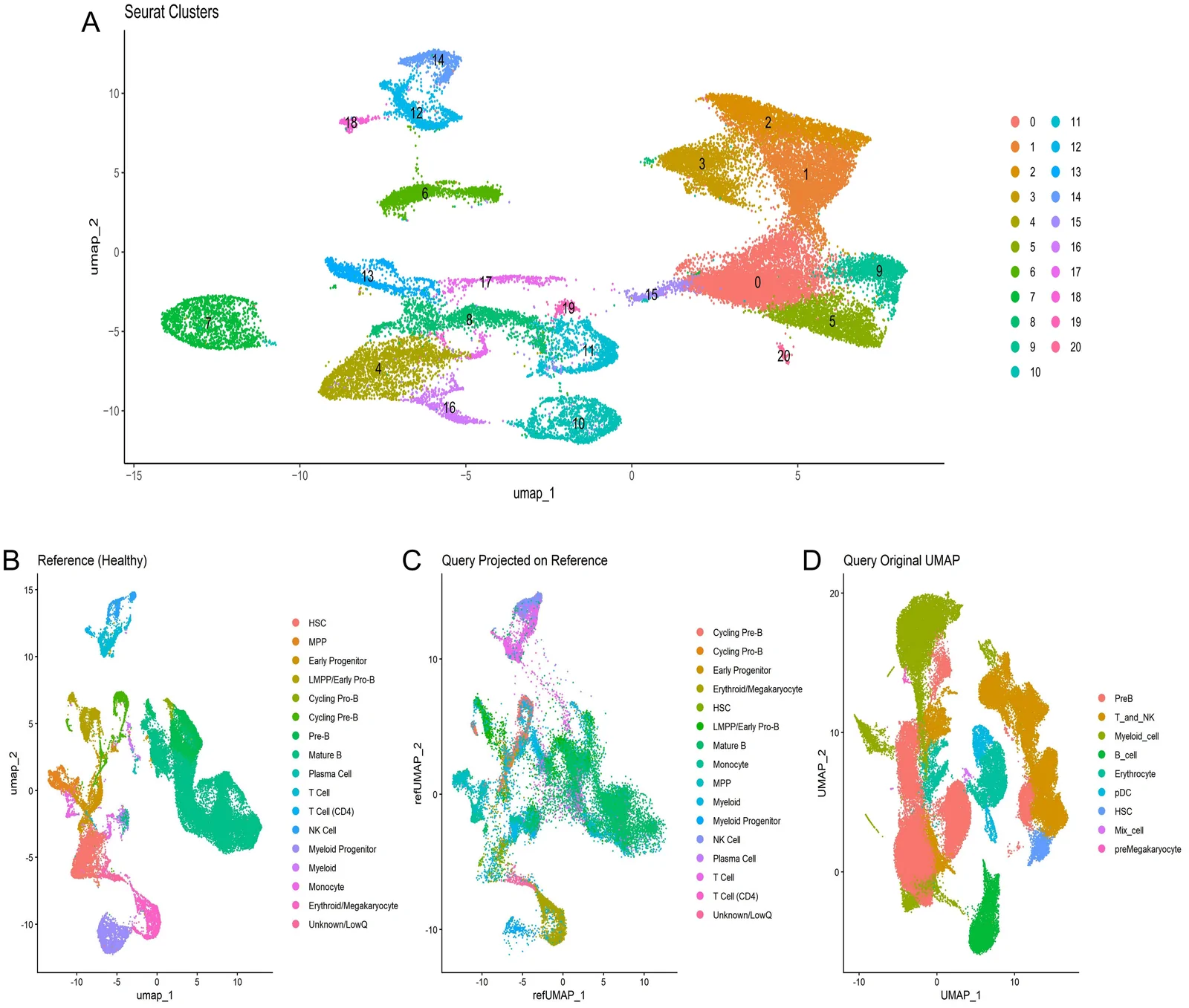
Single-cell RNA-sequencing atlas analysis of bone marrow hematopoietic cells in pediatric B-ALL. **(A)** Unsupervised UMAP clustering of normal human bone marrow cells generated by the Seurat algorithm. **(B)** UMAP visualization of the healthy human bone marrow reference atlas, annotated with major hematopoietic lineages, including hematopoietic stem cells (HSCs), multipotent progenitors (MPPs), B-cell subsets at different developmental stages, T cells, natural killer (NK) cells, myeloid cells and erythroid cells. **(C)** UMAP plot showing cell-type annotation transfer by projecting pediatric B-ALL patient cells onto the healthy reference atlas using Seurat v4 MapQuery. **(D)** Unsupervised UMAP clustering of B-ALL patient bone marrow cells performed independently without the healthy reference atlas.

The established reference atlas of healthy human bone marrow covered a full spectrum of hematopoietic lineages and diverse cell types, including hematopoietic stem cells (HSCs), multipotent progenitors (MPPs), myeloid/erythroid progenitors, monocytes, T/natural killer (NK) cells and B-cell subsets across different developmental stages. **Figure 2A** shows unsupervised clustering of normal bone marrow cells, and **Figure 2B** presents the corresponding cell type annotation results. Cell identities were validated using canonical lineage-specific marker genes: *CD34* and *FLT3* for HSCs/MPPs; *DNTT*, *MME*, *VPREB1* and *IGLL1* for pro-B/pre-B cells; *MS4A1* for mature B cells; and *MKI67* and *TOP2A* for proliferating B cells (**Supplementary Figure 1**). The top 10 marker genes of each cell cluster were visualized by a heatmap in **Supplementary Figure 3**, which further confirmed the accuracy of cell classification. We next performed unsupervised UMAP clustering solely on patient-derived single-cell data (**Figure 2D**), which revealed massive expansion of proliferative pro-B and pre-B leukemic blasts at diagnosis, accompanied by a marked reduction in normal hematopoietic populations. Subsequently, patient datasets were projected onto the UMAP space of the healthy bone marrow atlas, and cell annotation was implemented via the MapQuery pipeline (**Figure 2C**). Further projection analysis showed that leukemic blasts were predominantly located in the early B-cell developmental region and highly overlapped with normal pro-B/pre-B cells, indicating that these malignant cells were B-cell precursors arrested at an early developmental stage. In addition, the small populations of residual normal T cells, NK cells, monocytes and mature B cells in patient bone marrow were precisely matched to corresponding subsets in healthy controls, which fully verified the reliability of cell annotation and atlas projection in this study.

**Figure 3:**
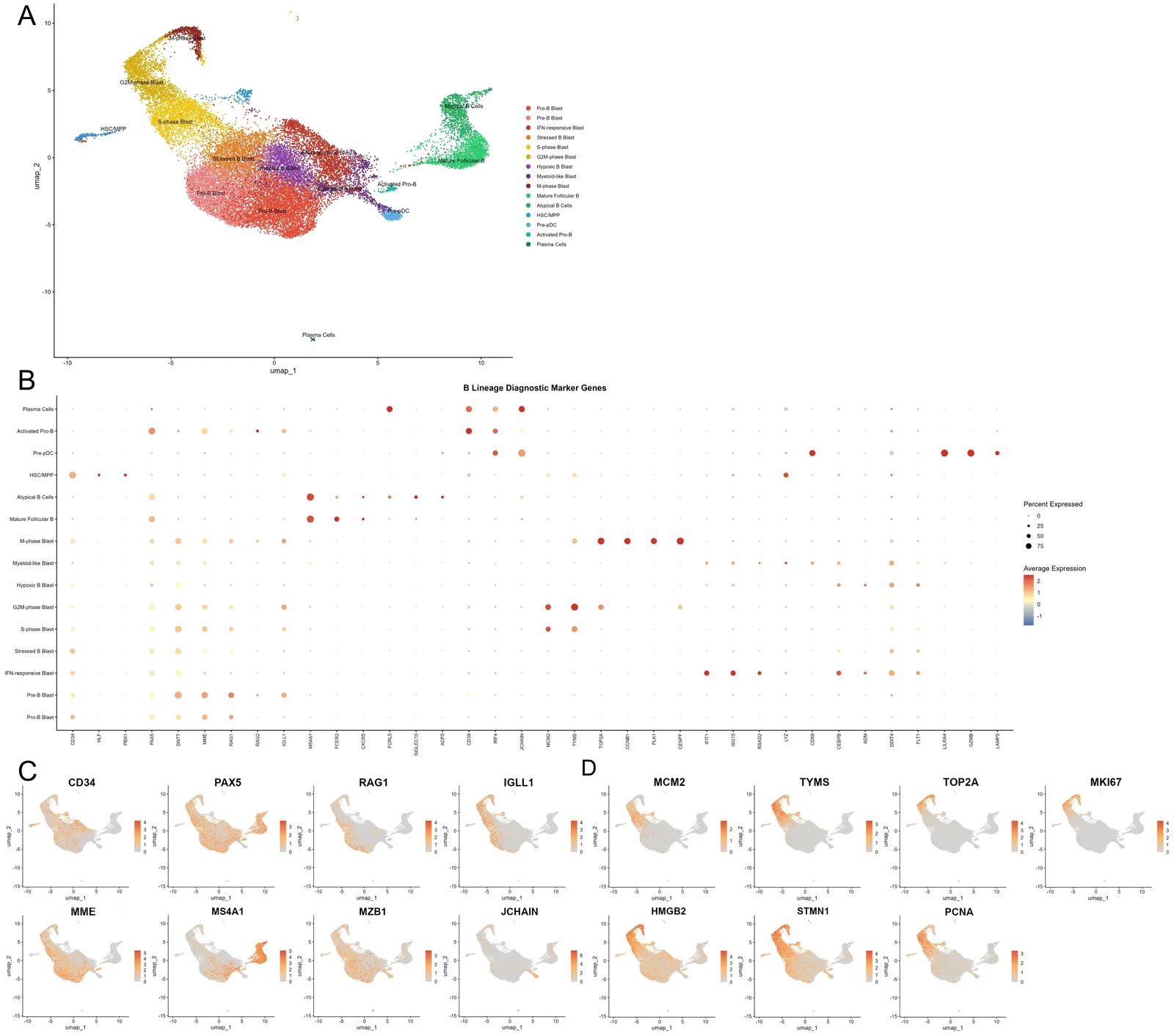
Fine clustering and gene expression characteristics of B-lineage cells in pediatric B-ALL patients. **(A)** UMAP visualization of 15 functionally defined subsets obtained by refined unsupervised clustering of B-lineage cells from pediatric B-ALL patients. **(B)** Dot plot showing the expression of canonical marker genes across B-lineage subsets, used to verify cell identities. Color intensity represents the average gene expression level, and dot size indicates the percentage of cells expressing the corresponding gene. **(C)** Feature plots showing expression patterns of key B-cell development marker genes (*CD34, PAX5, RAG1, IGLL1, MME, MS4A1, MZB1, JCHAIN*) across B-lineage cells. **(D)** Feature plots showing expression patterns of proliferation and cell cycle-related marker genes (*MKI67, PCNA, MCM2, TYMS, TOP2A, HMGB2, STMN1*) across B-lineage cells.

We further refined the single-cell transcriptomic data to systematically characterize B-lineage cell heterogeneity in pediatric B-ALL. After strict quality filtering, a total of 54,523 high-quality cells were retained from the initial 67,793 captured cells. Batch effects across 12 samples were eliminated, and the efficacy of data integration was validated by PCA plots before and after batch correction (**Supplementary Figure 4**). A total of 32 distinct cell clusters were identified via initial unsupervised clustering. To distinguish malignant blasts from normal B-lineage cells, CNV analysis was performed using InferCNV v3, with endogenous normal T cells serving as the reference. Guided by CNV profiles, B-lineage cells were re-clustered into 15 functionally distinct subsets (**Figure 3A**), and their identities were confirmed by a dot plot of signature gene expression (**Figure 3B**). CNV results confirmed that leukemic blasts dominated the bone marrow at diagnosis, with only a small number of normal B cells remaining. To delineate the developmental status and proliferative activity of B-lineage cells, we visualized the expression patterns of key genes associated with B-cell development and cell cycle progression (**Figures 3C, D**). Early progenitor markers *CD34* and *MME* (*CD10*) were predominantly expressed in leukemic blasts, while the pan-B cell transcription factor *PAX5* was universally detected in all B-lineage populations. Genes involved in early B-cell gene rearrangement, including *RAG1* and *IGLL1*, were enriched in pro-B/pre-B leukemic cells. In contrast, markers for mature B cells (*MS4A1/CD20*), marginal zone B cells (*MZB1*) and plasma cells (*JCHAIN*) were restricted to a minor fraction of residual normal cells. This stepwise expression pattern of lineage markers reflected a clear developmental hierarchy, demonstrating that the bone marrow microenvironment at diagnosis was largely occupied by immature leukemic blasts with developmental arrest. Notably, core proliferation-related genes (*MKI67*, *PCNA*, *MCM2*, *TYMS*, *TOP2A*, *HMGB2*, *STMN1*) were highly expressed in immature blasts, indicating that this subset represented the most proliferative compartment in the bone marrow. Mature B-lineage cells and most non-blast leukemic cells exhibited low proliferative activity and remained in a quiescent state.

**Figure 4:**
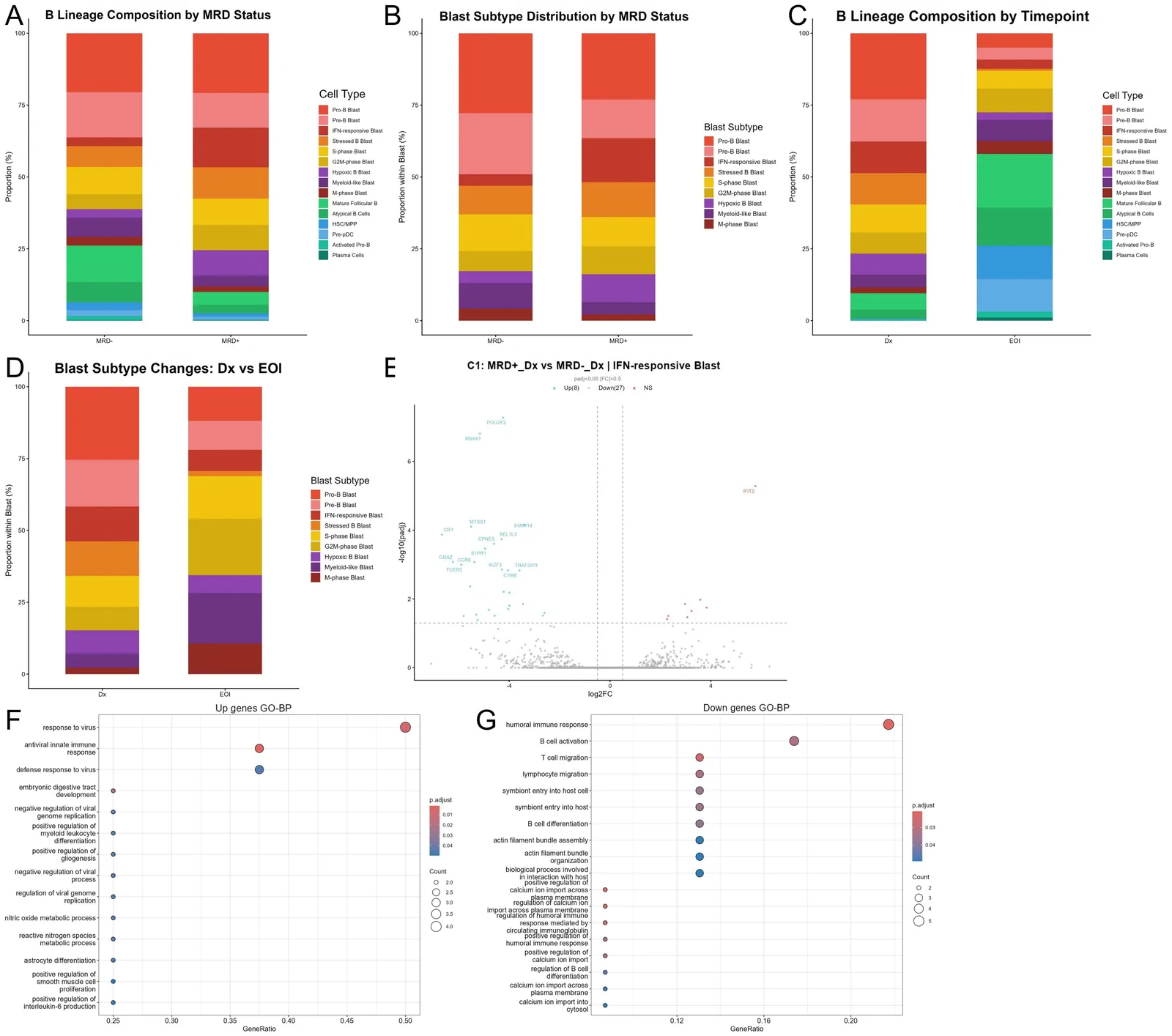
Dynamic changes of B-lineage cell composition and functional gene enrichment analysis in pediatric B-ALL patients with different MRD statuses and at different treatment stages. **(A, B)** Stacked bar charts showing the composition of B-lineage cell subsets **(A)** and the distribution of leukemic blast subsets **(B)** in MRD-negative and MRD-positive patients. **(C, D)** Stacked bar charts comparing the overall B-lineage cell composition **(C)** and leukemic blast subset distribution **(D)** between diagnosis (Dx) and the end of induction therapy (EOI). **(E)** Volcano plot of differentially expressed genes (DEGs) in IFN-responsive blasts between MRD-negative and MRD-positive patients. Genes with an adjusted P (FDR) value less than 0.05 and |log_2_FC| greater than 0.5 were defined as significantly DEGs. Blue dots represent upregulated genes, gray dots represent downregulated genes, and red dots indicate non-significantly changed genes (NS). **(F, G)** Gene Ontology biological process (GO-BP) enrichment analysis of upregulated **(F)** and downregulated **(G)** genes in IFN-responsive blasts from MRD-positive patients. The x-axis represents the GeneRatio (the ratio of DEGs to the total number of genes in each pathway), and the y-axis lists enriched biological process terms. Bubble color gradient corresponds to FDR-adjusted P value, with redder colors indicating higher enrichment significance. Bubble size represents the number of DEGs enriched in each pathway.

To explore the correlation between blast subsets and sustained MRD in B-ALL, we compared the composition of leukemic blasts between MRD-positive and MRD-negative patients. The relative proportions of blast subsets differed significantly between the two groups. MRD-positive patients presented markedly increased frequencies of IFN-responsive blasts and pre-B blasts, whereas the proportions of pro-B blasts and myeloid-like blasts were significantly decreased (**Figure 4A**). Stacked bar charts of B-lineage cell composition further confirmed these results, showing prominent enrichment of IFN-responsive blasts and profound depletion of normal B-cell populations in the bone marrow of MRD-positive patients (**Figure 4B**). These findings suggest that IFN-responsive blasts and early pre-B blasts are key cellular subsets driving persistent MRD in B-ALL.

We next focused on IFN-responsive blasts and profiled their unique molecular characteristics. Differential expression analysis identified 8 significantly upregulated genes and 27 downregulated genes in this cell subset (**Figure 4E**). Gene Ontology biological process (GO-BP) enrichment analysis revealed that upregulated genes were mainly enriched in pathways associated with inflammatory response and interferon signaling, suggesting that these cells are highly sensitive to pro-inflammatory stimuli within the bone marrow microenvironment. Bone marrow monocytes and macrophages are the major cellular sources of pro-inflammatory mediators, including type I interferons and interleukin-6 (IL-6). By comparison, downregulated genes were primarily involved in humoral immunity, B-cell activation and differentiation (**Figures 4F, G**), which indicates impaired B-cell maturation and defective adaptive immunity in IFN-responsive blasts. Taken together, IFN-responsive leukemic blasts in MRD-positive patients are characterized by excessive innate immune activation and blocked B-cell differentiation. This unique molecular phenotype is correlated with sustained MRD, and may represent a molecular feature associated with MRD persistence.

To clarify the remodeling effect of induction chemotherapy on B-lineage cells, we compared bone marrow B-cell compositions at two time points: Dx and EOI. Stacked bar plots revealed a dramatic shift in cellular components after treatment. The proportions of normal mature B cells, atypical B cells and hematopoietic stem/progenitor cells were substantially elevated, while the abundance of malignant leukemic blasts was sharply reduced (**Figure 4C**). Further subset analysis of leukemic blasts showed a comprehensive reprogramming of blast distribution from diagnosis to post-induction therapy (**Figure 4D**). At diagnosis, leukemic blasts were mainly composed of early developmental subsets (pro-B and pre-B blasts), along with abundant IFN-responsive blasts and highly proliferative blasts. After induction therapy, the frequencies of pro-B and pre-B blasts decreased obviously, and the residual MRD clones were predominantly IFN-responsive blasts and myeloid-like blasts. Cell cycle analysis demonstrated that blasts in the S and M phases were largely eliminated by chemotherapy, with a remarkable reduction in their relative proportions. Meanwhile, G2-phase blasts accumulated due to cell cycle arrest. Overall, the proportion of highly proliferative cells was much lower than that at diagnosis.

Dynamic analysis of bone marrow cellular composition indicated that induction chemotherapy preferentially eliminates highly proliferative leukemic blasts while a small fraction of IFN-responsive and myeloid-like blasts persist post-treatment. Leukemic blasts exhibit distinct proliferative heterogeneity, which correlates with composition of post-treatment residual lesions: fast-cycling immature blasts are highly susceptible to chemotherapy and effectively depleted during treatment. In contrast, cell cycle-arrested, quiescent IFN-responsive blasts display altered responses to interferon-mediated anti-leukemic stress. Although these blasts retain strong responsiveness to microenvironmental IFN signals, they may escape IFN-dependent anti-tumor immune clearance and utilize IFN signaling cascades to support cell survival, and are enriched among residual clones associated with persistent MRD. Consistent with this result, IFN-responsive blasts were significantly enriched in MRD-positive patients, confirming their critical role in sustaining residual disease and increasing relapse risk. Integrated clinical and single-cell analyses further verified that expanded non-classical monocytes at diagnosis act as an independent risk factor for MRD. This monocyte subset coincides with the formation of an IFN-rich bone marrow niche, which may support survival and drug tolerance in malignant blasts. Mechanistically, IFN-responsive blasts from MRD-positive patients show aberrant innate immune activation and arrested B-cell differentiation. By regulating anti-apoptotic pathways and immune checkpoint molecules, these cells exhibit enhanced survival potential and immune evasion features. Collectively, induction chemotherapy can effectively eradicate proliferative leukemic clones but fails to eliminate these drug-resistant blasts, which are enriched within residual populations linked to persistent MRD and subsequent disease relapse. Given the close clinical association between non-classical monocytes and chemo-resistant blast subsets, we hypothesized that extensive intercellular communication exists between these two cell populations in the bone marrow niche. Abundantly expanded non-classical monocytes may continuously secrete inflammatory cytokines and interferons to stabilize and reinforce the resistant phenotype of IFN-responsive blasts. To further dissect the regulatory mechanisms of MRD persistence, we performed cell-cell communication analysis to systematically characterize signaling interactions between non-classical monocytes and multiple blast subsets, including IFN-responsive blasts, pre-B blasts and pro-B blasts.

### 3.3 Functional heterogeneity of monocyte subsets and their crosstalk characteristics with leukemic blasts

**Figure 5:**
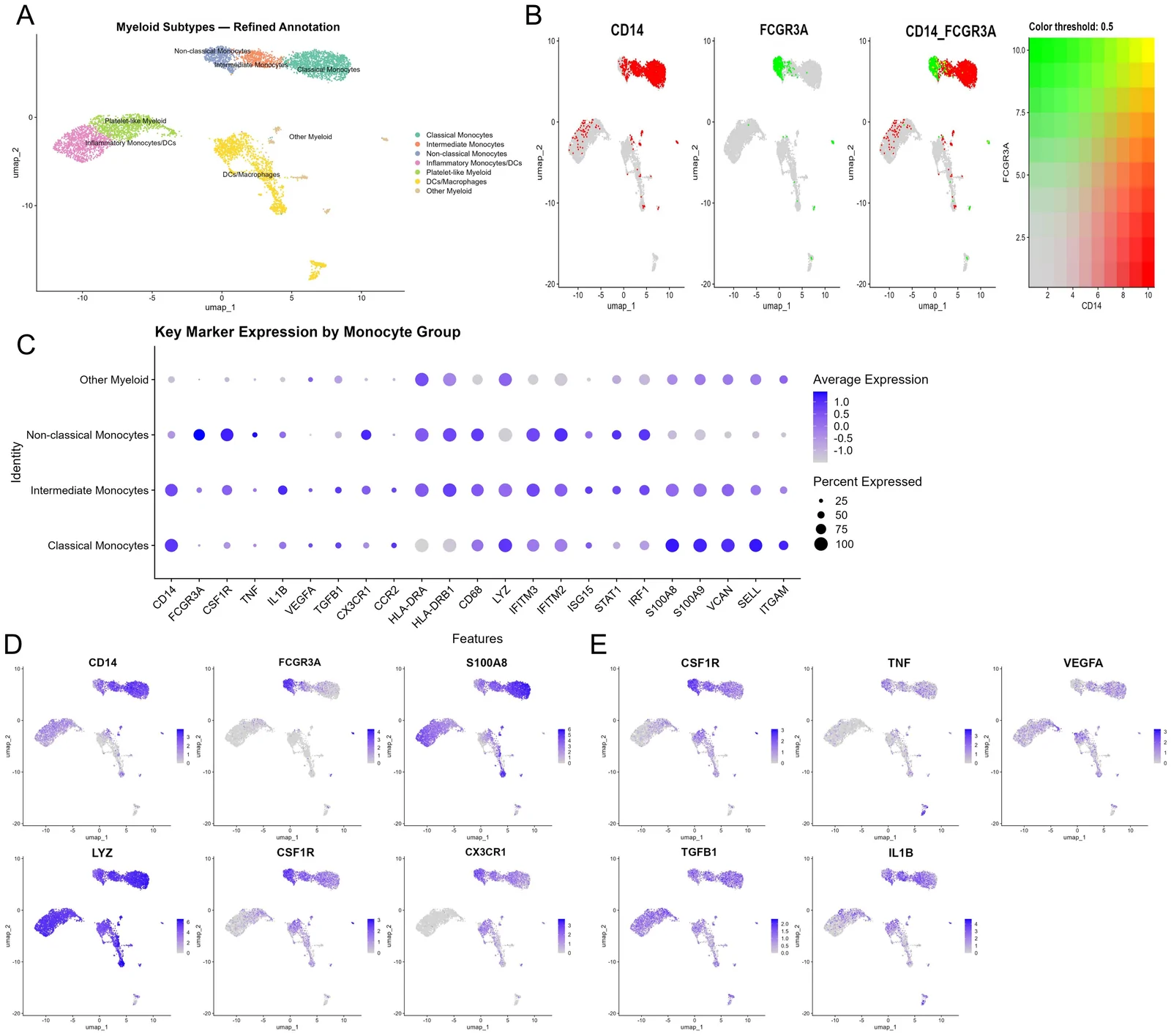
Refined annotation and gene expression profiling of myeloid and monocyte subsets in pediatric B-ALL patients. **(A)** UMAP visualization of myeloid cell populations after refined unsupervised clustering, showing seven distinct myeloid subsets: classical monocytes, intermediate monocytes, non-classical monocytes, inflammatory monocytes/dendritic cells (DCs), platelet-like myeloid cells, DCs/macrophages, and other myeloid cells. **(B)** Dual-gene expression UMAP plots for CD14 (red) and FCGR3A (green). The color gradient scale on the right illustrates their combined expression levels, confirming the characteristic CD14^low^FCGR3A^high^ phenotype of non-classical monocytes. **(C)** Dot plot showing the expression of key marker genes across monocyte subsets. Dot size represents the percentage of cells expressing the gene, and color intensity indicates the average expression level. **(D, E)** Feature plots showing the expression of canonical monocyte subset markers (*CD14, FCGR3A, S100A8, LYZ, CSF1R, CX3CR1*) **(D)** and functional genes (*CSF1R, TNF, VEGFA, TGFB1, IL1B*) **(E)**. Color intensity represents the normalized expression level of each gene.

Based on single-cell transcriptomic data of bone marrow samples from children with B-ALL, we performed refined unsupervised clustering on monocyte populations to systematically characterize the molecular features, functional heterogeneity and dynamic changes of monocyte subsets before and after chemotherapy. Three major monocyte subsets were identified: classical monocytes, intermediate monocytes and non-classical monocytes. Inflammatory monocytes/dendritic cells (DCs), platelet-like myeloid cells and DCs/macrophages were defined as control myeloid populations (**Figure 5A**). On the UMAP plot, the three monocyte subsets displayed a continuous distribution pattern, which is consistent with the stepwise differentiation and gradual functional transition of monocytes *in vivo*. Cell identities were validated using lineage-specific marker genes. Feature plots and dot plots revealed that classical monocytes highly expressed *CD14*, *S100A8* and *LYZ*. Non-classical monocytes exhibited a typical CD14^low^FCGR3A^high^ phenotype, with markedly elevated expression of *FCGR3A* (*CD16*) and *CX3CR1* (**Figures 5B–E**). Intermediate monocytes showed intermediate expression levels of these signature genes between classical and non-classical monocytes, indicating a transitional state during monocyte differentiation. Dual-gene co-expression analysis of *CD14* and *FCGR3A* further confirmed that non-classical monocytes formed an independent cluster with a stable CD14^low^FCGR3A^high^ profile in the UMAP space (**Figure 5B**). Functional enrichment analysis showed that non-classical monocytes were significantly enriched in genes associated with inflammatory response and angiogenesis, such as *TNF*, *IL1B* and *VEGFA*. These findings suggest that this subset possesses potent pro-inflammatory activity and the capacity to remodel the bone marrow microenvironment (**Figure 5E**).

**Figure 6:**
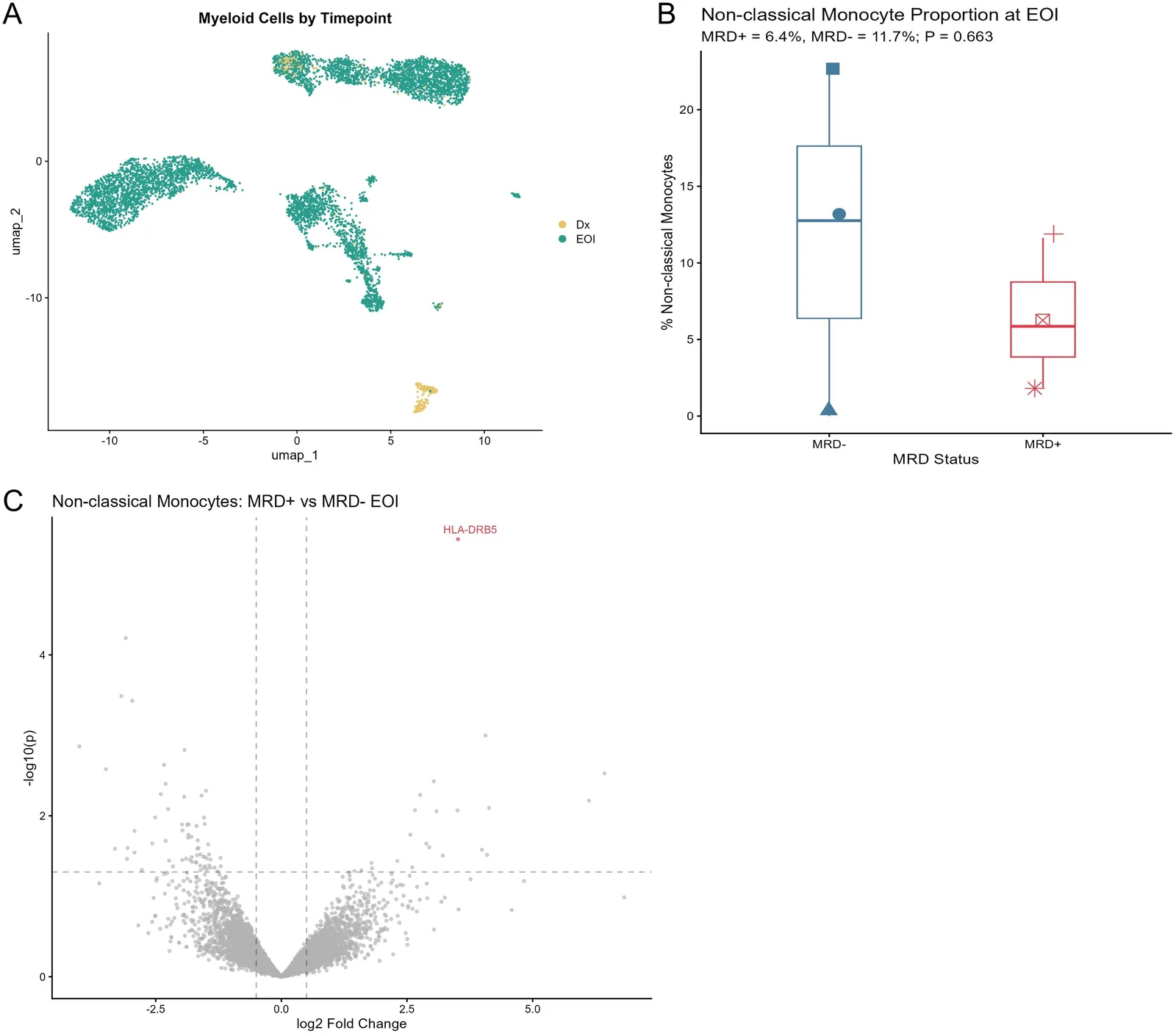
Temporal dynamics of myeloid cells and non-classical monocytes and their MRD-associated characteristics in pediatric B-ALL patients **(A)** UMAP visualization of myeloid cells colored by time points (Dx: diagnosis, yellow; EOI: end of induction, green). **(B)** Box plot comparing the proportion of non-classical monocytes between MRD-negative and MRD-positive patients at EOI. The mean percentage was 11.7% in MRD-negative patients versus 6.4% in MRD-positive patients (P = 0.663). **(C)** Volcano plot showing differentially expressed genes (DEGs) in non-classical monocytes between MRD-negative and MRD-positive patients at EOI. The x-axis represents log_2_ fold change, and the y-axis represents -log_10_(adjusted P value). The red dot highlights *HLA-DRB5*, the only significantly upregulated gene in MRD-positive patients.

To explore how chemotherapy reshapes monocyte subsets, we further analyzed dynamic alterations of bone marrow monocytes at the EOI therapy. UMAP clustering demonstrated obvious remodeling of monocyte composition after treatment compared with the baseline at diagnosis (**Figure 6A**). Quantification of monocyte subsets at EOI showed that the mean proportion of non-classical monocytes was 6.4% in MRD-positive patients versus 11.7% in MRD-negative patients. Although MRD-negative patients displayed a higher average abundance of non-classical monocytes at EOI, this disparity did not achieve statistical significance (P = 0.663) (**Figure 6B**). The observed trend toward fewer non-classical monocytes in MRD-positive individuals suggests that MRD status alone cannot independently regulate the quantitative recovery of non-classical monocytes after induction chemotherapy, yet it does not preclude the existence of biological links between residual leukemic blasts and monocyte subsets. To clarify functional differences in residual monocytes post-treatment, we conducted differential gene expression analysis of non-classical monocytes between the two groups (**Figure 6C**). Only *HLA-DRB5* was significantly upregulated in non-classical monocytes from the MRD-positive group, whereas no notable changes were observed in genes related to inflammatory activation or angiogenesis. As a key gene encoding major histocompatibility complex class II (MHC-II) molecule, elevated *HLA-DRB5* enhances the antigen-presenting capacity of non-classical monocytes, which may represent a compensatory immune response against residual leukemic blasts in MRD-positive patients (13–16). Previous studies on leukemia immune surveillance have demonstrated that persistent MRD can induce compensatory upregulation of MHC-II-related genes in myeloid antigen-presenting cells to strengthen anti-leukemic immunity (16). Nevertheless, this single transcriptional alteration fails to trigger global transcriptomic reprogramming in non-classical monocytes, which explains the lack of prominent changes in inflammation- and angiogenesis-related functional genes across groups. Collectively, although the abundance of non-classical monocytes differs significantly between MRD subgroups at EOI, these cells share highly consistent global transcriptomic profiles without obvious functional divergence.

**Figure 7:**
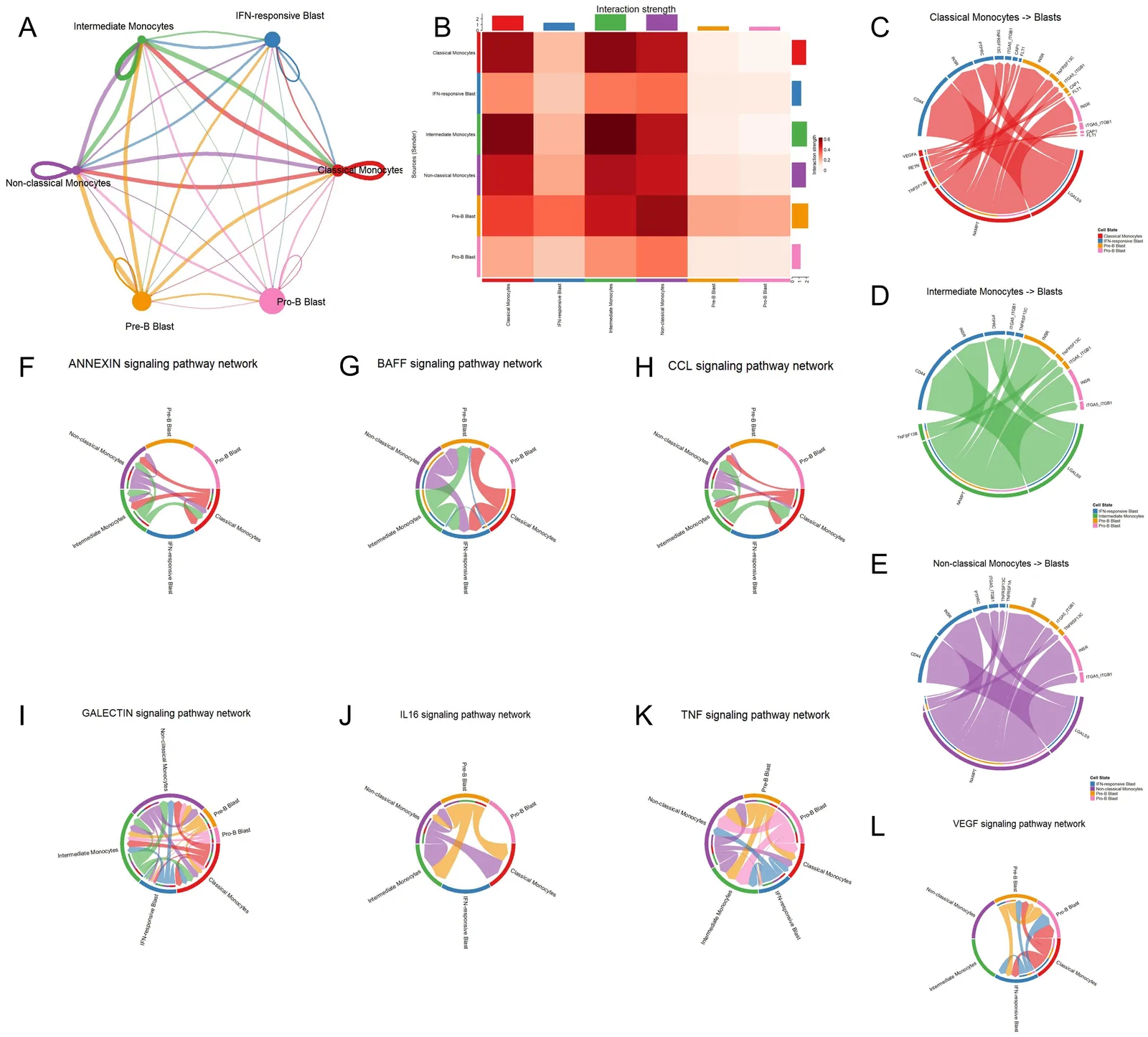
Cell-cell communication analysis between monocyte subsets and leukemic blasts in pediatric B-ALL patients. **(A)** Global cell-cell communication network generated by CellChat based on interaction weights, showing extensive signaling interactions among classical monocytes, intermediate monocytes, non-classical monocytes, and three types of leukemic blasts (Pro-B, Pre-B, and IFN-responsive blasts). Edge thickness corresponds to the intercellular communication strength between cell populations. **(B)** Heatmap showing the overall signaling communication strength between cell populations. Rows represent signaling sender cell groups, columns represent signaling receiver cell groups, and color intensity reflects the normalized interaction strength. **(C–E)** Circle plots illustrating ligand-receptor interactions from classical monocytes **(C)**, intermediate monocytes **(D)**, and non-classical monocytes **(E)** to leukemic blasts. Outer arcs represent cell subsets and their expressed ligands/receptors, and inner color bands represent intercellular interaction strength. **(F–L)** Interaction networks of specific signaling pathways: ANNEXIN **(F)**, BAFF [B-cell activating factor, **(G)**], CCL (chemokine, **H**), GALECTIN [galectin, **(I)**], IL16 [interleukin 16, **(J)**], TNF [tumor necrosis factor, **(K)**], and VEGF [vascular endothelial growth factor, **(L)**]. Line thickness indicates communication strength between different cell types.

Cell-cell communication is a core mechanism underlying the crosstalk between the bone marrow microenvironment and leukemic cells. Here, we used the CellChat platform to analyze ligand-receptor interactions and systematically delineate signaling patterns, differential ligand-receptor regulation and key pathways between monocyte subsets and leukemic blasts. Global cell communication networks based on interaction weights revealed extensive interactions among three monocyte subsets (classical, intermediate and non-classical monocytes) and three types of leukemic blasts (pro-B, pre-B and IFN-responsive blasts). Overall signaling was predominantly unidirectional, from leukemic blasts toward monocytes (**Figures 7A, B**). We next focused on reverse signaling derived from monocytes to leukemic blasts. All three monocyte subsets regulated cell adhesion, survival and immune functions via conserved ligand-receptor pairs, including LGALS9-galectin, CD44, INSR, TNFRSF13C and ITGA5-ITGB1. Notably, compared with classical and intermediate monocytes, non-classical monocytes displayed substantially enhanced pro-inflammatory and immunomodulatory signaling activities, accompanied by specific expression of *TNF* and *TNFSF13B*. This distinct feature highlights their unique regulatory roles in the bone marrow microenvironment (**Figures 7C, D**). Bone marrow monocytes can be recruited to sites harboring residual leukemic blasts. Through extensive ligand-receptor crosstalk, these immune cells participate in immune surveillance and microenvironment remodeling. Pathway enrichment analysis of cell communication identified major interacting pathways, including ANNEXIN, BAFF, CCL, GALECTIN, IL16, TNF and VEGF (**Figures 7E–L**). The GALECTIN pathway, which modulates cell adhesion and immune activation, was universally involved in interactions across all cell types and serves as a fundamental pathway sustaining microenvironmental communication. The TNF pathway was markedly activated in interactions involving non-classical monocytes, which is in line with their intrinsic pro-inflammatory transcriptomic signatures, suggesting that TNF signaling is a key pathway by which non-classical monocytes remodel the leukemic microenvironment. The classical chemotactic CCL pathway was mainly utilized by classical and intermediate monocytes to deliver chemotactic signals to leukemic blasts. In addition, the VEGF pathway acted as a unidirectional communication axis from monocytes to leukemic cells, potentially driving bone marrow angiogenesis and microenvironment remodeling after chemotherapy.

Taken together, we propose a model for MRD-associated microenvironment dysregulation in pediatric B-ALL. Under persistent stress imposed by residual leukemic blasts, monocytes are recruited to interact with tumor cells and remodel the bone marrow niche via widespread ligand-receptor crosstalk. Despite robust activation of intercellular inflammatory signaling, non-classical monocytes only exhibit compensatory upregulation of a single immune-related gene (*HLA-DRB5*), rather than global reprogramming of inflammation- or angiogenesis-associated transcripts. This limited immune compensatory signature correlates with impaired anti-leukemic immune surveillance and coincides with the presence of persistent MRD. From the perspective of overall regulatory networks, leukemic blasts exhibit widespread signaling interactions with monocytes and may contribute to altered monocyte phenotypic profiles. Heterogeneous monocyte subsets in turn exhibit distinct pro-inflammatory, chemotactic, and pro-survival signaling signatures, which are observed alongside disrupted bone marrow homeostasis and disease progression. The potent TNF and VEGF signaling activities of non-classical monocytes closely correlate with clinical MRD positivity and may represent a key molecular feature of MRD-associated microenvironmental changes. Notably, this is a single-center, retrospective exploratory study limited by a relatively small sample size. Further validation based on larger multicenter cohorts and *in vitro* functional experiments is required to verify these intercellular regulatory mechanisms and their potential for clinical translation.

## 4 Discussion

Utilizing clinical follow-up data combined with single-cell transcriptomic profiling, this study systematically explores the cellular and molecular mechanisms behind persistent MRD, chemoresistance and poor prognosis in pediatric B-ALL from three complementary perspectives: clinical prognostic factors, intrinsic leukemic heterogeneity, and monocyte-mediated remodeling of the immune microenvironment. Compared with conventional studies focusing only on routine clinical prognostic indicators, this multidimensional integrated analysis preliminarily identifies potential correlative signatures associated with disease progression in pediatric B-ALL. It also provides supportive evidence for optimizing prognostic stratification and developing therapeutic targets targeting the bone marrow microenvironment in clinical practice. Consistent with well-recognized molecular features of pediatric B-ALL, recurrent driver mutations including KRAS, NRAS, FLT3 and CREBBP detected in our cohort represent hallmark genetic alterations in pediatric B-ALL pathogenesis (17). Accumulating clinical and biological evidence further confirms that aberrant RAS pathway activation, predominantly induced by RAS family mutations, is tightly associated with impaired chemotherapy sensitivity and unfavorable long-term prognosis in B-ALL patients (18). Clinical prognostic analyses confirmed that EOI-MRD positivity was significantly associated with inferior EFS, which aligns with the well-established clinical consensus that post-induction MRD acts as a key auxiliary marker for evaluating treatment response and relapse risk (19). Notably, further multivariate Cox regression revealed a novel clinical observation: although conventional clinical risk stratification remained an independent prognostic predictor for long-term outcomes, EOI-MRD status failed to serve as an independent prognostic factor within our pediatric cohort. We acknowledge an important methodological limitation of this survival analysis: only 10 clinical endpoint events were recorded in the overall cohort. The low event count leads to limited statistical power in multivariate modeling, which likely contributes to the loss of independent prognostic significance for EOI-MRD after adjustment for baseline risk stratification. This finding indicates that despite the recognized prognostic value of MRD in general patient populations, combining traditional risk stratification with single time-point MRD measurement cannot fully explain interindividual variations in chemotherapeutic response and prognostic heterogeneity among real-world pediatric patients. Therefore, leukemic intrinsic heterogeneity and disrupted bone marrow immune homeostasis are indispensable supplementary dimensions for refining current prognostic systems and further deciphering the complex mechanisms of clinical chemoresistance. At the single-cell level, scRNA-seq profiling was performed on a limited cohort consisting of only 3 MRD-positive and 3 MRD-negative patients; preliminary transcriptomic observations suggested that conventional chemotherapy primarily eliminates highly proliferative leukemic blasts by inducing intracellular cytotoxic stress and irreversible cell damage. However, IFN-responsive leukemic subclones with blocked B-cell differentiation and stress-tolerant transcriptional signatures may withstand chemotherapy-induced cytotoxicity, evade treatment-related cell death, and persist stably after induction therapy. This preliminary observation partially explains the potential cellular origin of MRD in MRD-positive patients and reveals preliminary clues regarding the limitations of relying solely on MRD for prognostic assessment, rather than drawing definitive mechanistic conclusions. Our results further imply that intrinsic clonal heterogeneity is a core driver of chemoresistance, supporting the established theory of chemotherapy-mediated clonal selection. Specifically, dormant and stress-resistant leukemic subclones might survive cytotoxic injury, dominate the residual bone marrow niche, and eventually lead to sustained MRD and subsequent clinical relapse (20).

Dysregulated bone marrow immune microenvironment represents a critical extrinsic factor contributing to chemoresistance and persistent MRD in pediatric B-ALL. At diagnosis, patients with MRD positivity exhibit disrupted immune homeostasis, characterized by impaired T-cell immune surveillance, activation of an immunosuppressive niche, and specific accumulation of non-classical monocytes (7, 21). Notably, this association was observed in baseline clinical flow cytometry analyses, whereas intergroup differences in monocyte subsets were not detected in post-induction single-cell transcriptomic comparisons between MRD-positive and MRD-negative patients. Such temporal discrepancies may reflect profound immune remodeling induced by induction chemotherapy, which reshapes the composition and transcriptional profiles of bone marrow myeloid populations after treatment. In addition, this inconsistency may be partly constrained by the small sample size of our scRNA-seq cohort. Consistent with our single-cell data, chemotherapy spares IFN-responsive leukemic subclones with arrested differentiation that survive cytotoxic damage. Recruited non-classical monocytes migrate toward residual blasts and establish extensive ligand-receptor crosstalk. Distinct patterns of pathway usage underpin heterogeneous microenvironmental regulation across monocyte subsets. The Galectin pathway universally maintains cell adhesion and niche communication (22), while CCL chemokine signaling predominates in mediating cell recruitment and immune modulation in classical and intermediate monocytes (23). Uniquely, non-classical monocytes mainly engage TNF-driven pro-inflammatory signaling and VEGF-mediated vascular remodeling pathways (24, 25). Of note, extensive inflammatory crosstalk between cells does not trigger global transcriptomic reprogramming in these monocytes; instead, it only induces compensatory upregulation of *HLA-DRB5*. Through bidirectional communication with residual IFN-responsive leukemic subclones, non-classical monocytes are associated with a pro-inflammatory and pro-angiogenic bone marrow state that coincides with leukemic immune evasion, sustained cell survival and drug tolerance in blasts, alongside persistent MRD.

Integrated multidimensional analyses demonstrated that the development of MRD, chemoresistance and adverse clinical outcomes in pediatric B-ALL correlate with combined effects of clinical risk features, intrinsic leukemic clonal heterogeneity dominated by IFN-responsive subclones, and disrupted bone marrow immune homeostasis. Importantly, inflammatory disturbance and niche alterations linked to non-classical monocytes represent a potential correlative hub connecting clinical high-risk features to tumor-intrinsic drug tolerance, and coincide with the survival and persistence of residual IFN-responsive leukemic clones. Nevertheless, all above single-cell-derived mechanistic inferences should be interpreted cautiously due to the tiny sample size of only six patients for scRNA-seq detection, which restricts the generalizability and robustness of the relevant cellular and molecular conclusions. In summary, this study comprehensively provides preliminary clues to elucidates the potential mechanisms underlying MRD persistence and chemoresistance via integrated analyses of clinical data, cellular characteristics and microenvironmental changes, which advances our understanding of disease progression in pediatric B-ALL. These single-cell transcriptomic findings provide mechanistic evidence for optimizing clinical risk stratification, designing individualized therapeutic regimens, and developing microenvironment-targeted adjuvant therapies for pediatric B-ALL.

This study has several limitations. It is a single-center, retrospective exploratory investigation with a relatively small sample size. Notably, our scRNA-seq analysis only enrolled 3 MRD-positive and 3 MRD-negative patients, and such a limited sample cannot support solid, definitive mechanistic conclusions about IFN-responsive leukemic blasts and monocyte-mediated sustained MRD. Moreover, no *in vitro* or *in vivo* functional experiments were performed to validate the regulatory mechanisms of crosstalk between monocytes and leukemic cells. Future studies based on large-scale multicenter prospective cohorts, combined with *in vitro* co-culture systems, molecular intervention assays and *in vivo* animal models, are required to clarify the precise molecular mechanisms of monocyte-leukemia interactions and verify their clinical prognostic value. Such efforts will promote the clinical translation of therapies targeting the immune microenvironment for pediatric B-ALL.

## Data Availability

Data of this project can be accessed under accession codes CNP0009774 (single-cell sequencing data) and CNP0009776 (gene sequencing data) after an approval application to the China National Genebank (CNGB, https://db.cngb.org/cnsa/). Please refer to https://db.cngb.org/, or email: CNGBdb@cngb.org for detailed application guidance.
