# Supplementary material for: Interplay between monocyte subsets and leukemic blasts shapes the immune microenvironment and sustains minimal residual disease in pediatric B-cell acute lymphoblastic leukemia
Source: Front Immunol. 2026 Aug 18;17:1776564. doi: 10.3389/fimmu.2026.1776564 (PMC13529978; doi:10.3389/fimmu.2026.1776564)
Supplement: Supplementary file 1 [file SupplementaryFile1.docx]

Supplementary Material

# Supplementary Table 1 ：Baseline Clinical and Laboratory Characteristics of 6 Pediatric Patients with B-ALL in the Single-Cell Sequencing Cohort

| **Group** | **Sex** | **Age** | **WBC** | **ANC** | **ALC** | **AMC** | **AEC** | **ABC** | **Hb** | **PLT** | **ABL** | **ECOG PS** | **Bone Marrow Blast %** | **LDH** | **CNS Status** |
| --- | --- | --- | --- | --- | --- | --- | --- | --- | --- | --- | --- | --- | --- | --- | --- |
|  |  |  | **(×10⁹/L)** | **(×10⁹/L)** | **(×10⁹/L)** | **(×10⁹/L)** | **(×10⁹/L)** | **(×10⁹/L)** | **(g/L)** | **(×10⁹/L)** | **(×10⁹/L)** |  |  | **(U/L)** |  |
| MRD⁻ | F | 5 y | 70.5 | 0.8 | 57.5 | 1.6 | 0 | 0.1 | 78 | 22 | 10.58 | 1 | 77.2 | 512 | CNS1 |
| MRD⁻ | M | 6 y | 20.1 | 1.4 | 9.3 | 1 | 0.1 | 0 | 76 | 94 | 8.04 | 1 | 78.8 | 465 | CNS1 |
| MRD⁻ | F | 2 y 11 m | 3.1 | 0.2 | 2.8 | 0.1 | 0 | 0 | 70 | 31 | 0 | 1 | 56 | 238 | CNS1 |
| MRD⁺ | F | 11 y | 5.41 | 3.3 | 2 | 0.1 | 0.01 | 0.02 | 85 | 331 | 0 | 1 | 94 | 222 | CNS1 |
| MRD⁺ | M | 7 y | 7.7 | 0.5 | 4.4 | 0.3 | 0.1 | 0 | 91 | 54 | 2.31 | 1 | 90 | 440 | CNS1 |
| MRD⁺ | M | 2 y 7 m | 6.8 | 2.5 | 4.2 | 0.1 | 0 | 0 | 84 | 167 | 0 | 1 | 78.4 | 241 | CNS1 |

WBC, white blood cell count; ANC, absolute neutrophil count; ALC, absolute lymphocyte count; AMC, absolute monocyte count; AEC, absolute eosinophil count; ABC, absolute basophil count; Hb, hemoglobin; PLT, platelet count; LDH, lactate dehydrogenase; CNS, central nervous system; F, female; M, male.

**Supplementary Figure 1**

**Supplementary Figure 1.** Dot plot showing the expression of representative lineage-specific marker genes in normal bone marrow cell subsets. The dot size and color represent the percentage of expressed cells and the average expression level, respectively.

## Supplementary Figure 2

**Supplementary Figure 2.** Quality control analysis of single-cell RNA-sequencing data. UMAP plots show the distribution of UMI count (nCount_RNA), gene number (nFeature_RNA), and mitochondrial gene percentage (percent.mt) in all single cells.

**Supplementary Figure 3**

**Supplementary Figure 3.** Heatmap displaying the top 10 signature marker genes of each cell cluster in the healthy bone marrow reference atlas.

**Supplementary Figure 4**

**Supplementary Figure 4.** Batch effect correction of 12 single-cell RNA-sequencing samples from pediatric B-ALL patients and healthy donors. PCA plots show the integration effect before and after batch correction, indicating reliable removal of batch differences and high consistency of the integrated dataset.
